# Rich-Club Organization Disturbances of the Individual Morphological Network in Subjective Cognitive Decline

**DOI:** 10.3389/fnagi.2022.834145

**Published:** 2022-02-25

**Authors:** Liling Peng, Jing Feng, Di Ma, Xiaowen Xu, Xin Gao

**Affiliations:** ^1^Shanghai Universal Medical Imaging Diagnostic Center, Shanghai, China; ^2^The Fifth People’s Hospital of Jinan, Jinan, China; ^3^College of Information Science and Technology, Nanjing Forestry University, Nanjing, China; ^4^Department of Medical Imaging, School of Medicine, Tongji Hospital, Tongji University, Shanghai, China

**Keywords:** subjective cognitive decline, rich-club, Alzheimer’s disease, morphological network, gray matter (GM)

## Abstract

**Background:**

Subjective cognitive decline (SCD) was considered to be the preclinical stage of Alzheimer’s disease (AD). However, less is known about the altered rich-club organizations of the morphological networks in individuals with SCD.

**Methods:**

This study included 53 individuals with SCD and 54 well-matched healthy controls (HC) from the Alzheimer’s disease Neuroimaging Initiative (ADNI) database. Individual-level brain morphological networks were constructed by estimating the Jensen-Shannon distance-based similarity in the distribution of regional gray matter volume. Rich-club properties were then detected, followed by statistical comparison.

**Results:**

The characteristic rich-club organization of morphological networks (normalized rich-club coefficients > 1) was observed for both the SCD and HC groups under a range of thresholds. The SCD group showed a reduced normalized rich-club coefficient compared with the HC group. The SCD group exhibited the decreased strength and degree of rich-club connections than the HC group (strength: HC = 79.93, SCD = 74.37, *p* = 0.028; degree: HC = 85.28, SCD = 79.34, *p* = 0.027). Interestingly, the SCD group showed an increased strength of local connections than the HC group (strength: HC = 1982.16, SCD = 2003.38, *p* = 0.036).

**Conclusion:**

Rich-club organization disturbances of morphological networks in individuals with SCD reveal a distinct pattern between the rich-club and peripheral regions. This altered rich-club organization pattern provides novel insights into the underlying mechanism of SCD and could be used to investigate prevention strategies at the preclinical stage of AD.

## Introduction

Alzheimer’s disease (AD) is a progressive neurodegenerative disease characterized by amyloid plaques (Aβ) and neurofibrillary tangles in gray matter (GM). Subjective cognitive decline (SCD) has been recognized as a risk in individuals who have subjective memory complaints but have no evidence of objective cognitive impairment. It might be the preclinical stage of AD and increase the risk of conversion to mild cognitive impairment (MCI) and AD (Jessen et al., 2014; Buckley et al., 2016). Recent neuroimaging studies indicated that SCD has exhibited an abnormal pattern of functional and structural network disruptions, which are similar to the alterations in MCI and AD (Chen et al., 2020; Gao et al., 2020; Xu et al., 2021). However, it is largely unknown whether the individuals with SCD show the altered topological organization of the morphological networks.

The nervous system is characterized by a complex network that makes up the “connectome,” which supports complex cognition. The neurological dysfunction may originate from abnormal topological properties of the brain network. Most studies of brain networks are conducted using resting-state functional magnetic resonance imaging (rs-fMRI) and diffusion tensor imaging (DTI), and individual-level brain morphological networks based on the 3D-T1 MRI have been developed in recent years and their application in exploring disorder mechanisms are limited (Wang et al., 2016). Recent studies have demonstrated the existence of rich-club organization (highly connected brain regions connecting preferentially to other important regions), which plays a key role in integrating information transmission (Van Den Heuvel and Sporns, 2011; Li et al., 2017). Emerging evidence has suggested that AD and MCI have significant hub-concentrated lesion distributions (Kim et al., 2019; Li et al., 2021b). However, Daianu et al. (2015) proposed that the disruption of the white matter network was predominant in the peripheral brain network in AD. Besides rs-fMRI and DTI, structural MRI (sMRI) has attracted increased attention in exploring whole-brain morphological connectivity patterns. Very few studies have been done on the club organization of morphological networks in individuals with SCD.

In this study, we used sMRI to construct individual whole-brain morphological networks and further investigated the alterations in the rich-club organization in individuals with SCD as compared with healthy elderly. Based on previous findings of disrupted functional and structural connectivity in AD, we hypothesized that altered topological properties of the morphological networks can be detected as early as the SCD stage.

## Materials and Methods

### Subjects

Data used in this research were obtained from the Alzheimer’s disease Neuroimaging Initiative (ADNI) database,^1^ and all subjects included in this study were from the ADNI-2 and ADNI-3. The primary goal of the ADNI has been to test whether neuropsychological assessment, neuroimaging, and biological markers could be combined to track the progression of AD. For up-to-date information, see http://www.adni-info.org. Appropriate Institutional Review Boards approval was sought at each ADNI site, and informed consent was obtained from each participant.

In this study, we included 53 subjects with SCD and 54 sex-, age-, and education-matched healthy controls (HC) from the ADNI database. The detailed diagnostic criteria were described in the ADNI manual. Briefly, HC participants had no subjective or informant-reported memory decline and showed normal cognitive performance on the Mini-Mental State Examination (MMSE, between 24 and 30), a Clinical Dementia Rating (CDR, score = 0), and the Wechsler Memory Scale Logical Memory (WMS-LM) delayed recall (adjusted for education level); SCD participants showed subjective memory concerns as evaluated using the Cognitive Change Index (CCI; total score from the first 12 items ≥ 16), normal cognitive performance on the MMSE, CDR, and WMS-LM delayed recall and no informant-reported complaint of memory decline. We excluded subjects with significant medical, neurological, and psychiatric illnesses. For example, we excluded the subjects with clinical depression using the geriatric depression scale-15 (GDS-15 score > 5). Each participant was assessed using a standardized clinical evaluation protocol that included the MMSE; WMS-LM immediate and delayed recall; Rey Auditory Verbal Learning Test (RAVLT) total, immediate, and delayed recall; Trail-Making Test Parts A and B (TMT-A and -B); category fluency—animals test; and clock-drawing test (CDT). In Table 1, we presented the detailed demographics and neuropsychological data of the participants.

**TABLE 1:** Demographic and neuropsychological data.

Items	HC (*n* = 54)	SCD (*n* = 53)	Statistical value	*p-*value
Age (years)	74.02 ± 6.93	72.08 ± 6.23	1.52	0.13^b^
Education (years)	16.54 ± 2.12	17.08 ± 2.20	–1.29	0.20^b^
Gender (male/female)	32/22	33/20	0.75	0.84^a^
Neuropsychiatric Scores				
CCI	32/54 (13.84 ± 1.25)	53/53 (22.49 ± 6.52)	–7.40	< 0.001^b^*
General mental status				
MMSE	28.83 ± 1.63	28.89 ± 1.25	–0.19	0.85^b^
Cognitive subdomain scores				
WMS-LM immediate recall	15.04 ± 3.47	14.96 ± 3.25	0.12	0.91^b^
WMS-LM delayed recall	14.04 ± 3.63	13.58 ± 3.54	0.65	0.52^b^
RAVLT total	47.64 ± 9.78	45.69 ± 10.17	1.00	0.32^b^
RAVLT immediate recall	9.68 ± 3.63	9.70 ± 3.15	–0.03	0.98^b^
RAVLT delayed recall	8.79 ± 3.91	8.72 ± 4.49	0.09	0.93^b^
TMT-A	29.33 ± 7.99	31.04 ± 9.82	–0.99	0.33^b^
TMT-B	72.76 ± 37.30	76.68 ± 39.63	–0.53	0.60^b^
Category fluency-Animals	22.07 ± 5.98	22.57 ± 6.08	–0.42	0.67^b^
CDT	4.69 ± 0.51	4.47 ± 0.59	–0.48	0.64^b^

*Values are presented as the mean ± standard deviation (SD).*

*^a^The p-value was obtained by χ^2–^test.*

*^b^The p-value was obtained by two-sample t-tests.*

* indicates a statistical difference between groups, p < 0.05.

*HC, health controls; SCD, subjective cognitive decline; MMSE, Mini-Mental State Examination; CCI, Cognitive Change Index; WMS-LM, Wechsler Memory Scale Logical Memory; RAVLT, Rey Auditory Verbal Learning Test; TMT, Trail-Making Test; CDT, Clock Drawing Test.*

### Structural Magnetic Resonance Imaging Acquisition

All structural MRI scans were downloaded from ADNI for the participants. All participants were examined using a SIEMENS 3.0T scanner. Scans were corrected before download as previously described (Nudelman et al., 2014).

### Image Preprocessing and Network Construction

All imaging data preprocessing were carried out using the Computational Anatomy Toolbox (CAT12)^2^ based on Statistical Parametric Mapping 12 (SPM12).^3^ First, the structural 3D T1-weighted images were segmented into GM, white matter, and cerebrospinal fluid with the default parameters. The resultant GM images were subsequently normalized to the Montreal Neurological Institute (MNI) space using a high-dimensional approach and further non-linearly modulated to compensate for spatial normalization effects. After these steps, a GM volume map was obtained for each participant (a voxel size of 1.5 × 1.5 × 1.5 mm). Spatial smoothing is a typically used step to increase the signal-to-noise ratio. We performed the following analyses separately for GM volume maps with spatial smoothing (Gaussian kernel with 6-mm full width at half maximum) (Wang et al., 2016).

**FIGURE 1:**
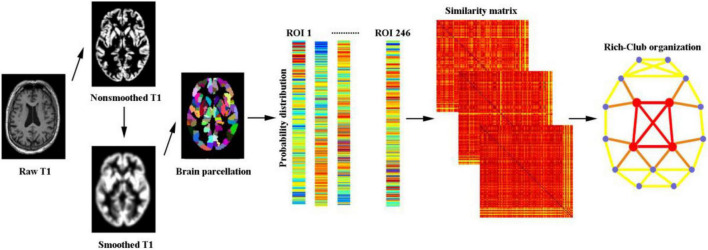
A flowchart illustrating the construction of morphological networks in this study.

For the network nodes, the human Brainnetome Atlas was used to divide the whole brain into 246 regions of interest (ROIs) (the abbreviations are listed in Supplementary Table 1). For morphological networks, we utilized a Jensen-Shannon distance-based similarity (JSS) to quantify the morphological connectivity between two regions (Endres and Schindelin, 2003; Li et al., 2021a). For each participant, the GM volume values of all voxels in each ROI were first extracted; subsequently, the kernel density estimation was used to estimate the probability density function of these GM volume values. Then, the probability distribution function was calculated based on the obtained probability density function. Afterward, the JSS value between any pair of ROIs was calculated based on their probability distribution function, which ranges from 0 to 1. A higher JSS value was obtained when the GM density distribution of two ROIs was closer. Notably, there are some KL-based methods. Specifically, the benefits of Jensen-Shannon divergence similarity estimation (JSSE) are two sides compared with the KL-based methods. First, the range of JSS divergence is (0,1), resulting in a more accurate judgment of the similarity. Second, the JSS divergence is symmetrical, which makes it easier to portray the connections between ROIs. We applied a set of sparsity thresholds (ranging from 0.1 to 0.4, with steps of 0.01) to generate a binary undirected network. In Figure 1, this range of sparsity thresholds was chosen because networks were not fully connected at lower sparsity thresholds and were less likely to maintain small-world architecture at higher sparsity thresholds (Chen et al., 2019).

### Rich-Club Organization

The rich-club coefficient was originally proposed to quantify the connectivity density between high-degree nodes (i.e., hubs) in a network (Colizza et al., 2006). For a binary network, the rich-club coefficient Φ(*k*) is calculated as the ratio of the total number of connections among a specific set of nodes with a degree (degree is defined as the number of edges that directly link to a given node) larger than *k* divided by the maximum possible number of connections among this set of nodes. Φ(*k*) was normalized relative to the Φ_random_(*k*) of a set of comparable random networks (*n* = 1,000) of equal size and degree sequence, yielding a normalized rich-club coefficients Φ_norm_(*k*) = Φ(*k*)/Φ_random_(*k*). Φ_norm_(*k*) > 1 over a range of degrees (*k*) indicates the existence of a rich-club organization in the brain connectome. The rich-club analyses were based on the GRETNA toolbox.^4^

The hub regions were selected based on the average cortical network across the HC group and defined as the top 25 (10%) brain regions with the highest degree (Xu et al., 2020). Once the nodes were classified as hub nodes and peripheral nodes, the edges of the network were classified as rich-club connections between two hub nodes, feeder connections from one hub node to one peripheral node, or local connections between two peripheral nodes.

### Statistical Analysis

Analyses were performed using the Statistical Package for Social Sciences (SPSS, Version 22). Demographic factors and clinical scores including age, years of education, gender, and cognitive scores were compared between the HC group and SCD group. Gender distribution was compared using the Chi-square test. Age, years of education, and cognitive performance between the HC group and SCD group were compared using a two-sample *t*-test. The significance level was set at *p* < 0.05.

At the sparsity thresholds (ranging from 0.1 to 0.4, with steps of 0.01), normalized rich-club coefficients were compared using a two-sample *t*-test. To compare the abnormal connections in the SCD group and HC group, a two-sample *t*-test was used (*p* < 0.001, uncorrected).

## Results

### Demographic and Clinical Characteristics

Demographic and clinical data for the HC group and the SCD group are summarized in Table 1. No significant differences were found in the age and gender between the HC and SCD groups. The SCD group displayed significantly increased CCI scores compared with the HC group (*p* < 0.001). Additionally, there were no significant differences in cognitive performance.

### Group Differences in Rich-Club Organization

**FIGURE 2:**
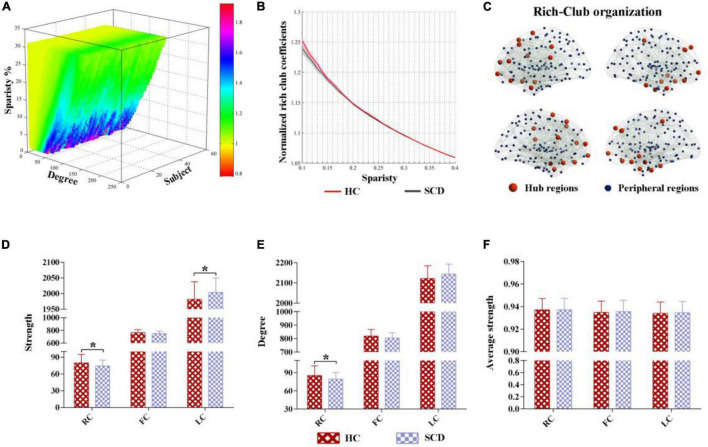
The altered rich-club organization of morphological networks between SCD and HC. **(A)** The characteristic rich-club organization of morphological networks (normalized rich-club coefficients > 1) was observed for both the SCD and HC groups ranging from the sparsity of 0.1–0.4. **(B)** The biggest difference of normalized rich-club coefficients was observed in the sparsity threshold of 0.1 (HC = 1.25, SCD = 1.23). **(C)** The top 25 (10%) highest-degree nodes were defined as hub regions and the remaining 221 regions were classified as peripheral regions. **(D–F)** Significant differences in the strength and degree of the rich-club and local connections were identified, while no significant differences were found in the average strength between the HC and SCD groups. SCD, subjective cognitive decline; HC, healthy controls. * indicates a statistical difference between groups, *p* < 0.05.

The characteristic rich-club organization of morphological networks (normalized rich-club coefficients > 1) was observed for both the SCD and HC groups under a range of thresholds (ranging from 0.1 to 0.4) (Figures 2A,B). The SCD group showed reduced normalized rich-club coefficients compared with the HC group from the sparsity of 0.1–0.4. However, there were no significant statistical differences of normalized rich-club coefficients between the two groups ranging from the sparsity of 0.1–0.4. In addition, the biggest difference of normalized rich-club coefficients was observed in the sparsity threshold of 0.1 (HC = 1.25, SCD = 1.23) (Figure 2B). Thus, the connectivity analysis in the rich-club organization was based on the network density at 10% for each subject.

Based on the average nodal degree across the HC group, we identified the hub regions by sorting the nodal degrees. The top 25 (10%) highest-degree nodes were defined as hub nodes and were primarily distributed in the prefrontal, lateral temporal, and medial parietal regions, which was consistent with the findings from previous studies (Van Den Heuvel and Sporns, 2011). The remaining 221 regions were classified as peripheral nodes, as shown in Figure 2C. Moreover, significant differences in the strength and degree of the rich-club and local connections were identified, while no significant differences were found in the average strength between the HC and SCD groups (Figures 2D–F). In detail, the SCD group exhibited the decreased strength and degree of rich-club connections than the HC group (strength: HC = 79.93, SCD = 74.37, *p* = 0.028; degree: HC = 85.28, SCD = 79.34, *p* = 0.027) (Figures 2D,E). Interestingly, the SCD group showed an increased strength of local connections than the HC group (strength: HC = 1,982.16, SCD = 2,003.38, *p* = 0.036) (Figure 2F).

### Group Differences in Morphological Connectivity

**FIGURE 3:**
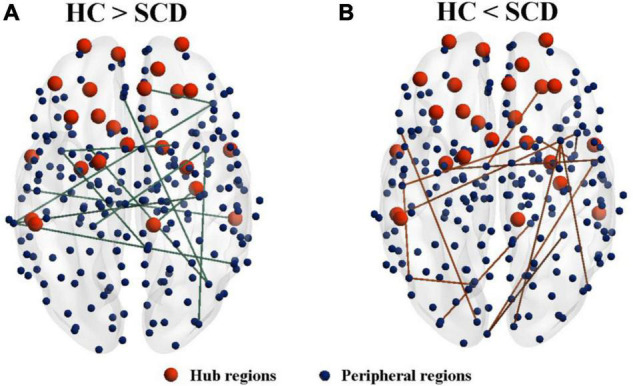
The altered connected subnetwork is based on the connectome analysis. **(A)** A disrupted connected network with 12 connections was altered in the SCD group compared with the HC group (*p* < 0.001, uncorrected), comprising 1 rich-club connection, 2 feeder connections, and 9 local connections. **(B)** Compared with the HC group, the SCD group showed the enhanced network composed of 24 nodes and 15 edges (*p* < 0.001, uncorrected), comprising 1 feeder connection and 14 local connections. SCD, subjective cognitive decline; HC, healthy controls.

Network analysis revealed abnormal connectivity in the morphological networks for individuals with SCD (Table 2). A disrupted connected network with 12 connections was altered in the SCD group compared with the HC group (*p* < 0.001, uncorrected), comprising 1 rich-club connection, 2 feeder connections, and 9 local connections (Figure 3A). However, compared with the HC group, the SCD group showed the enhanced network composed of 24 nodes and 15 edges (*p* < 0.001, uncorrected), comprising 1 feeder connection and 14 local connections (Figure 3B).

**TABLE 2:** The abnormal connectivity between the HC and SCD groups.

Connectivity			HC (mean value)	SCD (mean value)	*p*-value
Connections	Region A	Region B			
	**HC > SCD**				
	OrG_R_6_6	SFG_R_7_6	0.89	0.85	< 0.001
	STG_L_6_4	OrG_R_6_6	0.85	0.81	< 0.001
	STG_L_6_4	PrG_R_6_3	0.84	0.81	< 0.001
	FuG_R_3_1	PCL_R_2_1	0.90	0.86	< 0.001
	IPL_R_6_5	STG_L_6_4	0.83	0.78	< 0.001
	ITG_R_7_7	ITG_L_7_4	0.88	0.84	< 0.001
	CG_L_7_3	IPL_R_6_2	0.83	0.79	< 0.001
	Str_L_6_4	IPL_R_6_2	0.85	0.82	< 0.001
	INS_L_6_6	PCun_R_4_2	0.83	0.78	< 0.001
	INS_R_6_6	PoG_L_4_4	0.85	0.79	< 0.001
	Str_R_6_4	INS_L_6_6	0.83	0.80	< 0.001
	OcG_R_4_1	INS_R_6_6	0.82	0.78	< 0.001
	**HC < SCD**				
	OrG_R_6_3	SFG_L_7_5	0.87	0.90	< 0.001
	STG_L_6_2	MFG_R_7_2	0.84	0.89	< 0.001
	ITG_R_7_3	MFG_R_7_2	0.85	0.90	< 0.001
	PrG_R_6_2	MFG_R_7_6	0.84	0.88	< 0.001
	Cun_R_5_3	MFG_R_7_6	0.83	0.88	< 0.001
	OcG_R_4_2	MFG_R_7_6	0.82	0.86	< 0.001
	sOcG_L_2_1	IFG_L_6_6	0.85	0.89	< 0.001
	PhG_L_6_1	PrG_R_6_1	0.78	0.81	< 0.001
	IPL_L_6_5	STG_L_6_3	0.83	0.87	< 0.001
	Cun_L_5_3	STG_R_6_3	0.81	0.85	< 0.001
	OcG_L_4_4	PhG_R_6_6	0.83	0.87	< 0.001
	Cun_L_5_3	SPL_R_5_3	0.80	0.86	< 0.001
	Cun_L_5_5	IPL_L_6_5	0.83	0.87	< 0.001
	Str_R_6_4	PoG_L_4_1	0.79	0.81	< 0.001
	Str_R_6_4	INS_R_6_4	0.82	0.84	< 0.001

*HC, health controls; SCD, subjective cognitive decline.*

## Discussion

In this study, we investigated the rich-club organization of single-subject brain morphological networks in individuals with SCD. We found that the SCD group exhibited different variation patterns in the rich-club structure. In detail, compared with the HC group, the SCD group showed less strength in the rich-club connections but more strength in the local connections.

Our results revealed that the morphological networks of SCD showed significantly decreased rich-club connections. Brain network abnormalities may be more concentrated on rich-club regions, which may be caused by a hub-focused biology and more easily detectable by neuroimaging approaches (Crossley et al., 2014). A growing body of research has focused on the rich-club organization from the perspectives of functional MRI (fMRI) and diffusion MRI networks in the AD spectrum disease. Very few studies have focused on the topology of the morphological networks in SCD. An fMRI study has found that the SCD group remained relatively stable in the rich-club organization, but the rich-club coefficient of morphological networks has shown a downward trend in our study (Xue et al., 2020). Our finding is consistent with previous studies that the functional disconnection of MCI and AD were mainly located in highly connected brain hubs (Dai et al., 2019; Li et al., 2020). Wang et al. (2021) combined the fMRI and diffusion MRI networks to explore the rich-club organization in AD. They found that the functional brain network, but not the structural brain network, showed reduced rich-club connections in AD. Some previous studies have also indicated that AD had relatively preserved rich-club connections in the diffusion brain network (Daianu et al., 2016).

However, from the perspective of morphological networks, abnormal morphological connectivity in rich-club regions emerges before disruptions in the diffusion network become apparent in the preclinical phase of AD. This pattern is consistent with a typical AD deterioration processing with abnormal Aβ accumulation occurring first, followed sequentially by the disruption of the function and structure of neurons, and, finally, disturbance of the axons and synapses. The synapse loss could be caused by the failure of live neurons to maintain the normal function of axons and dendrites or by neuron death (Bloom, 2014). Intriguingly, a recent DTI study has found that the individuals with SCD showed reduced connectivity involving peripheral regions but remained stable in rich-club regions (Yan et al., 2018). Another DTI study has indicated that the SCD group exhibited less connection strength including the rich-club, feeder, and local connections (Shu et al., 2018). Longitudinal studies of the same study population are needed to further demonstrate the stability and reliability of the imaging biomarkers.

Although rich-club connections of morphological networks were significantly decreased, increased local connections were found in individuals with SCD. Dai et al. (2019) demonstrated that AD showed the increased couplings between functional and structural networks in rich-club structure, which may imply a more strengthened relationship between functional and white matter connectivity in AD. The hub node is also consistent with the existing studies (Van Den Heuvel et al., 2012; Collin et al., 2017; Liang et al., 2018). More interestingly, another multimodal neuroimaging study has indicated that increases in the functional-structural connectivity coupling of feeder and local connections were also found in patients with MCI and AD (Cao et al., 2020). A previous multimodal connectome study has shown that the SCD group exhibited an increased morphological connectivity between the right superior parietal lobe and orbital gyrus, which was consistent with the local connection in our study (Chen et al., 2021). We speculate that the higher morphological connections between the peripheral regions may represent the compensatory recruitment to maintain normal cognitive performance in individuals with SCD. These findings and our results enhanced the understanding of the underlying neural mechanisms of AD spectrum disease by different MRI modalities from the perspective of rich-club organization.

## Limitations

This study has a few limitations. First, our study was a cross-sectional observational study and had a relatively small sample size. The longitudinal follow-up studies in a population-based cohort are needed to validate the results. Second, the different parcellation strategies could affect the topological organization of the brain network. Third, the optimal sparsity range may vary from the sample size, and the data-driven based topological filtering techniques, such as orthogonal minimal spanning trees (OMST) (Dimitriadis et al., 2017) and efficiency cost optimisation (ECO) (Luppi et al., 2021), will be considered in our future work. Moreover, other brain templates need to further assess the reliability of the rich-club organization. Finally, we only focused on the morphological networks. The combination of multimodal neuroimaging may yield a comprehensive understanding of the underlying mechanisms in SCD.

## Conclusion

We proposed that rich-club organization disturbances of morphological networks in SCD imply a distinct pattern between the rich club and peripheral regions. This altered rich-club organization pattern provides novel insights into the underlying mechanism of SCD and could be used to investigate prevention strategies for patients with early AD.

## Data Availability Statement

The original contributions presented in the study are included in the article/Supplementary Material, further inquiries can be directed to the corresponding author/s.

## Author Contributions

LP and JF drafted the initial manuscript. DM and XG collected and pre-processed the fMRI data. XX designed experiments and analyzed the results. XG and XX revised the manuscript. All authors contributed to the article and approved the submitted version.

## Conflict of Interest

The authors declare that the research was conducted in the absence of any commercial or financial relationships that could be construed as a potential conflict of interest.

## Publisher’s Note

All claims expressed in this article are solely those of the authors and do not necessarily represent those of their affiliated organizations, or those of the publisher, the editors and the reviewers. Any product that may be evaluated in this article, or claim that may be made by its manufacturer, is not guaranteed or endorsed by the publisher.

## Footnotes

1
http://adni.loni.usc.edu

2
http://www.neuro.uni-jena.de/cat/

3
http://www.fil.ion.ucl.ac.uk/spm/software/spm12/

4
http://www.nitrc.org/projects/gretna/

## References

[B1] Bloom G. S. (2014). Amyloid-β and tau: the trigger and bullet in Alzheimer disease pathogenesis. *JAMA Neurol.* 71 505–508. 10.1001/jamaneurol.2013.5847 24493463 PMC12908160

[B2] Buckley R. F. Maruff P. Ames D. Bourgeat P. Martins R. N. Masters C. L. (2016). Subjective memory decline predicts greater rates of clinical progression in preclinical Alzheimer’s disease. *Alzheimer’s Dementia* 12 796–804. 10.1016/j.jalz.2015.12.013 26852195

[B3] Cao R. Wang X. Gao Y. Li T. Zhang H. Hussain W. (2020). Abnormal anatomical rich-club organization and structural–functional coupling in mild cognitive impairment and Alzheimer’s disease. *Front. Neurol.* 11:53. 10.3389/fneur.2020.00053 32117016 PMC7013042

[B4] Chen H. Huang L. Yang D. Ye Q. Guo M. Qin R. (2019). Nodal global efficiency in front-parietal lobe mediated periventricular white matter hyperintensity (Pwmh)-related cognitive impairment. *Front. Aging Neurosci.* 11:347. 10.3389/fnagi.2019.00347 31920627 PMC6914700

[B5] Chen H. Li W. Sheng X. Ye Q. Zhao H. Xu Y. (2021). Machine learning based on the multimodal connectome can predict the preclinical stage of Alzheimer’s disease: a preliminary study. *Eur. Radiol.* 32 448–459. 10.1007/s00330-021-08080-9 34109489

[B6] Chen H. Sheng X. Luo C. Qin R. Ye Q. Zhao H. (2020). The compensatory phenomenon of the functional connectome related to pathological biomarkers in individuals with subjective cognitive decline. *Transl. Neurodegen.* 9 1–14. 10.1186/s40035-020-00201-6 32460888 PMC7254770

[B7] Colizza V. Flammini A. Serrano M. A. Vespignani A. (2006). Detecting rich-club ordering in complex networks. *Nat. Phys.* 2 110–115. 10.1038/nphys209

[B8] Collin G. Scholtens L. H. Kahn R. S. Hillegers M. H. Van Den Heuvel M. P. (2017). Affected anatomical rich club and structural–functional coupling in young offspring of schizophrenia and bipolar disorder patients. *Biol. Psychiatry* 82 746–755. 10.1016/j.biopsych.2017.06.013 28734460

[B9] Crossley N. A. Mechelli A. Scott J. Carletti F. Fox P. T. Mcguire P. (2014). The hubs of the human connectome are generally implicated in the anatomy of brain disorders. *Brain* 137 2382–2395. 25057133 10.1093/brain/awu132 PMC4107735

[B10] Dai Z. Lin Q. Li T. Wang X. Yuan H. Yu X. (2019). Disrupted structural and functional brain networks in Alzheimer’s disease. *Neurobiol. Aging* 75 71–82. 10.1016/j.neurobiolaging.2018.11.005 30553155

[B11] Daianu M. Jahanshad N. Nir T. M. Jack C. R. Jr. Weiner M. W. Bernstein M. A. (2015). Rich club analysis in the Alzheimer’s disease connectome reveals a relatively undisturbed structural core network. *Hum. Brain Mapp.* 36 3087–3103. 10.1002/hbm.22830 26037224 PMC4504816

[B12] Daianu M. Mezher A. Mendez M. F. Jahanshad N. Jimenez E. E. Thompson P. M. (2016). Disrupted rich club network in behavioral variant frontotemporal dementia and early-onset A lzheimer’s disease. *Hum. Brain Mapp.* 37 868–883. 10.1002/hbm.23069 26678225 PMC4883024

[B13] Dimitriadis S. I. Antonakakis M. Simos P. Fletcher J. M. Papanicolaou A. C. (2017). Data-driven topological filtering based on orthogonal minimal spanning trees: application to multigroup magnetoencephalography resting-state connectivity. *Brain Connect.* 7 661–670. 10.1089/brain.2017.0512 28891322 PMC6435350

[B14] Endres D. M. Schindelin J. E. (2003). A new metric for probability distributions. *IEEE Trans. Inform. Theory* 49 1858–1860.

[B15] Gao X. Xu X. Hua X. Wang P. Li W. Li R. (2020). Group similarity constraint functional brain network estimation for Mild Cognitive Impairment classification. *Front. Neurosci.* 14:165. 10.3389/fnins.2020.00165 32210747 PMC7076152

[B16] Jessen F. Amariglio R. E. Van Boxtel M. Breteler M. Ceccaldi M. Chételat G. (2014). A conceptual framework for research on subjective cognitive decline in preclinical Alzheimer’s disease. *Alzheimer’s Dementia* 10 844–852. 10.1016/j.jalz.2014.01.001 24798886 PMC4317324

[B17] Kim G. H. Kim J. Y. Kim J. E. Ma J. Kim B. R. Im J. J. (2019). Alterations in structural rich-club connectivity of the precuneus are associated with depressive symptoms among individuals with subjective memory complaints. *Cogn. Affect. Behav. Neurosci.* 19 73–87. 10.3758/s13415-018-0645-x 30298425

[B18] Li W. Tang Y. Wang Z. Hu S. Gao X. (2021a). The reconfiguration pattern of individual brain metabolic connectome for Parkinson’s disease identification. *arXiv [preprint]* 10.1002/mco2.305 PMC10300308 37388240

[B19] Li W. Xu X. Wang Z. Peng L. Gao X. Wang P. (2021b). Multiple connection pattern combination from single-mode data for mild cognitive impairment identification. *Front. Cell Dev. Biol.* 9:782727. 10.3389/fcell.2021.782727 34881247 PMC8645991

[B20] Li W. Wang Z. Zhang L. Qiao L. Shen D. (2017). Remodeling Pearson’s correlation for functional brain network estimation and autism spectrum disorder identification. *Front. Neuroinform.* 11:55. 10.3389/fninf.2017.00055 28912708 PMC5583214

[B21] Li W. Xu X. Jiang W. Wang P. Gao X. (2020). Functional connectivity network estimation with an inter-similarity prior for mild cognitive impairment classification. *Aging (Albany Ny)* 12:17328. 10.18632/aging.103719 32921634 PMC7521542

[B22] Liang X. Hsu L.-M. Lu H. Sumiyoshi A. He Y. Yang Y. (2018). The rich-club organization in rat functional brain network to balance between communication cost and efficiency. *Cerebral Cortex* 28 924–935. 10.1093/cercor/bhw416 28108494 PMC6059143

[B23] Luppi A. I. Gellersen H. M. Peattie A. R. Manktelow A. E. Menon D. K. Dimitriadis S. I. (2021). Searching for consistent brain network topologies across the garden of (Shortest) forking paths. *bioRxiv [preprint]* 10.1101/2021.07.13.452257

[B24] Nudelman K. N. Risacher S. L. West J. D. Mcdonald B. C. Gao S. Saykin A. J. (2014). Association of cancer history with Alzheimer’s disease onset and structural brain changes. *Front. Physiol.* 5:423. 10.3389/fphys.2014.00423 25400589 PMC4215790

[B25] Shu N. Wang X. Bi Q. Zhao T. Han Y. (2018). Disrupted topologic efficiency of white matter structural connectome in individuals with subjective cognitive decline. *Radiology* 286 229–238. 10.1148/radiol.2017162696 28799862

[B26] Van Den Heuvel M. P. Kahn R. S. GoñI J. Sporns O. (2012). High-cost, high-capacity backbone for global brain communication. *Proc. Natl. Acad. Sci. U.S.A.* 109 11372–11377. 10.1073/pnas.1203593109 22711833 PMC3396547

[B27] Van Den Heuvel M. P. Sporns O. (2011). Rich-club organization of the human connectome. *J. Neurosci.* 31 15775–15786. 10.1523/JNEUROSCI.3539-11.2011 22049421 PMC6623027

[B28] Wang B. Wang G. Wang X. Cao R. Xiang J. Yan T. (2021). Rich-club analysis in adults with Adhd connectomes reveals an abnormal structural core network. *J. Attention Disord.* 25 1068–1079. 10.1177/1087054719883031 31640493

[B29] Wang H. Jin X. Zhang Y. Wang J. (2016). Single-subject morphological brain networks: connectivity mapping, topological characterization and test–retest reliability. *Brain Behav.* 6:e00448. 10.1002/brb3.448 27088054 PMC4782249

[B30] Xu X. Li W. Mei J. Tao M. Wang X. Zhao Q. (2020). Feature selection and combination of information in the functional brain connectome for discrimination of mild cognitive impairment and analyses of altered brain patterns. *Front. Aging Neurosci.* 12:28. 10.3389/fnagi.2020.00028 32140102 PMC7042199

[B31] Xu X. Wang T. Li W. Li H. Xu B. Zhang M. (2021). Morphological, structural, and functional networks highlight the role of the cortical-subcortical circuit in individuals with subjective cognitive decline. *Front. Aging Neurosci.* 13:688113. 10.3389/fnagi.2021.688113 34305568 PMC8299728

[B32] Xue C. Sun H. Hu G. Qi W. Yue Y. Rao J. (2020). Disrupted patterns of rich-club and diverse-club organizations in subjective cognitive decline and amnestic mild cognitive impairment. *Front. Neurosci.* 14:575652. 10.3389/fnins.2020.575652 33177982 PMC7593791

[B33] Yan T. Wang W. Yang L. Chen K. Chen R. Han Y. (2018). Rich club disturbances of the human connectome from subjective cognitive decline to Alzheimer’s disease. *Theranostics* 8:3237. 10.7150/thno.23772 29930726 PMC6010989

