# Supplementary material for: Rich-Club Organization Disturbances of the Individual Morphological Network in Subjective Cognitive Decline
Source: Front Aging Neurosci. 2022 Feb 25;14:834145. doi: 10.3389/fnagi.2022.834145 (PMC8914315; doi:10.3389/fnagi.2022.834145)
Supplement: Supplementary file 1 [file Table_1.docx]

**Supplemental Table 1**

| **Supplemental Table 1 Brain areas and their abbreviations in Brainnetome Atlas** | | | | | |
| --- | --- | --- | --- | --- | --- |
| **Lobe** | **Gyrus** | **Left and Right Hemisphere** | **Lobe** | **Gyrus** | **Left and Right Hemisphere** |
| **Frontal Lobe** | SFG, Superior Frontal Gyrus | SFG_L(R)7_1 | **Parietal Lobe** | SPL, Superior Parietal Lobule | SPL_L(R)5_1 |
|  |  | SFG_L(R)7_2 |  |  | SPL_L(R)5_2 |
|  |  | SFG_L(R)7_3 |  |  | SPL_L(R)5_3 |
|  |  | SFG_L(R)7_4 |  |  | SPL_L(R)5_4 |
|  |  | SFG_L(R)7_5 |  |  | SPL_L(R)5_5 |
|  |  | SFG_L(R)7_6 |  | IPL, Inferior Parietal Lobule | IPL_L(R)6_1 |
|  |  | SFG_L(R)7_7 |  |  | IPL_L(R)6_2 |
|  | MFG, Middle Frontal Gyrus | MFG_L(R)7_1 |  |  | IPL_L(R)6_3 |
|  |  | MFG_L(R)7_2 |  |  | IPL_L(R)6_4 |
|  |  | MFG_L(R)7_3 |  |  | IPL_L(R)6_5 |
|  |  | MFG_L(R)7_4 |  |  | IPL_L(R)6_6 |
|  |  | MFG_L(R)7_5 |  | Pcun, Precuneus | PCun_L(R)4_1 |
|  |  | MFG_L(R)7_6 |  |  | PCun_L(R)4_2 |
|  |  | MFG_L(R)7_7 |  |  | PCun_L(R)4_3 |
|  | IFG, Inferior Frontal Gyrus | IFG_L(R)6_1 |  |  | PCun_L(R)4_4 |
|  |  | IFG_L(R)6_2 |  | PoG, Postcentral Gyrus | PoG_L(R)4_1 |
|  |  | IFG_L(R)6_3 |  |  | PoG_L(R)4_2 |
|  |  | IFG_L(R)6_4 |  |  | PoG_L(R)4_3 |
|  |  | IFG_L(R)6_5 |  |  | PoG_L(R)4_4 |
|  |  | IFG_L(R)6_6 | **Insular Lobe** | INS, Insular Gyrus | INS_L(R)6_1 |
|  | OrG, Orbital Gyrus | OrG_L(R)6_1 |  |  | INS_L(R)6_2 |
|  |  | OrG_L(R)6_2 |  |  | INS_L(R)6_3 |
|  |  | OrG_L(R)6_3 |  |  | INS_L(R)6_4 |
|  |  | OrG_L(R)6_4 |  |  | INS_L(R)6_5 |
|  |  | OrG_L(R)6_5 |  |  | INS_L(R)6_6 |
|  |  | OrG_L(R)6_6 | **Limbic Lobe** | CG, Cingulate Gyrus | CG_L(R)7_1 |
|  | PrG, Precentral Gyrus | PrG_L(R)6_1 |  |  | CG_L(R)7_2 |
|  |  | PrG_L(R)6_2 |  |  | CG_L(R)7_3 |
|  |  | PrG_L(R)6_3 |  |  | CG_L(R)7_4 |
|  |  | PrG_L(R)6_4 |  |  | CG_L(R)7_5 |
|  |  | PrG_L(R)6_5 |  |  | CG_L(R)7_6 |
|  |  | PrG_L(R)6_6 |  |  | CG_L(R)7_7 |
|  | PCL, Paracentral Lobule | PCL_L(R)2_1 | **Occipital Lobe** | Cun, Cuneus Gyrus | Cun_L(R)5_1 |
|  |  | PCL_L(R)2_2 |  |  | Cun _L(R)5_2 |
| **Temporal Lobe** | STG, Superior Temporal Gyrus | STG_L(R)6_1 |  |  | Cun _L(R)5_3 |
|  |  | STG_L(R)6_2 |  |  | Cun _L(R)5_4 |
|  |  | STG_L(R)6_3 |  |  | Cun _L(R)5_5 |
|  |  | STG_L(R)6_4 |  | OcG, Occipital Gyrus | OcG_L(R)4_1 |
|  |  | STG_L(R)6_5 |  |  | OcG _L(R)4_2 |
|  |  | STG_L(R)6_6 |  |  | OcG _L(R)4_3 |
|  | MTG, Middle Temporal Gyrus | MTG_L(R)4_1 |  |  | OcG_L(R)4_4 |
|  |  | MTG_L(R)4_2 |  | sOcG, Superior Occipital Gyrus | sOcG _L(R)2_1 |
|  |  | MTG_L(R)4_3 |  |  | sOcG _L(R)2_2 |
|  |  | MTG_L(R)4_4 | **Subcortical Nuclei** | Amyg, Amygdala | Amyg_L(R)2_1 |
|  | ITG, Inferior Temporal Gyrus | ITG_L(R)7_1 |  |  | Amyg_L(R)2_2 |
|  |  | ITG_L(R)7_2 |  | Hipp, Hippocampus | Hipp_L(R)2_1 |
|  |  | ITG_L(R)7_3 |  |  | Hipp_L(R)2_2 |
|  |  | ITG_L(R)7_4 |  | Str, Striatum | Str_L(R)6_1 |
|  |  | ITG_L(R)7_5 |  |  | Str_L(R)6_2 |
|  |  | ITG_L(R)7_6 |  |  | Str_L(R)6_3 |
|  |  | ITG_L(R)7_7 |  |  | Str_L(R)6_4 |
|  | FuG, Fusiform Gyrus | FuG_L(R)3_1 |  |  | Str_L(R)6_5 |
|  |  | FuG_L(R)3_2 |  |  | Str_L(R)6_6 |
|  |  | FuG_L(R)3_3 |  | Tha, Thalamus | Tha_L(R)8_1 |
|  | PhG, Parahippocampal Gyrus | PhG_L(R)6_1 |  |  | Tha_L(R)8_2 |
|  |  | PhG_L(R)6_2 |  |  | Tha_L(R)8_3 |
|  |  | PhG_L(R)6_3 |  |  | Tha_L(R)8_4 |
|  |  | PhG_L(R)6_4 |  |  | Tha_L(R)8_5 |
|  |  | PhG_L(R)6_5 |  |  | Tha_L(R)8_6 |
|  |  | PhG_L(R)6_6 |  |  | Tha_L(R)8_7 |
|  | pSTS, posterior Superior Temporal Sulcus | pSTS_L(R)2_1 |  |  | Tha_L(R)8_8 |
|  |  | pSTS_L(R)2_2 |  | | |
